# Factors Affecting the Power Conversion Efficiency in ZnO DSSCs: Nanowire vs. Nanoparticles

**DOI:** 10.3390/ma11030411

**Published:** 2018-03-09

**Authors:** Myrsini Giannouli, Κaterina Govatsi, George Syrrokostas, Spyros N. Yannopoulos, George Leftheriotis

**Affiliations:** 1 Renewable Energy and Environment Laboratory, Physics Department, University of Patras, Rion GR-26500, Greece; glefther@physics.upatras.gr; 2 Foundation for Research and Technology Hellas, Institute of Chemical Engineering Sciences (FORTH/ICE-HT), P.O. Box 1414, Rio-Patras GR-26504, Greece; govatsi@iceht.forth.gr (K.G.); gsirrokostas@yahoo.gr (G.S.); sny@iceht.forth.gr (S.N.Y.)

**Keywords:** dye-sensitized solar cells, ZnO semiconductor, dye loading, nanowire, pH modification

## Abstract

A comparative assessment of nanowire versus nanoparticle-based ZnO dye-sensitized solar cells (DSSCs) is conducted to investigate the main parameters that affect device performance. Towards this aim, the influence of film morphology, dye adsorption, electron recombination and sensitizer pH on the power conversion efficiency (PCE) of the DSSCs is examined. Nanoparticle-based DSSCs with PCEs of up to 6.2% are developed and their main characteristics are examined. The efficiency of corresponding devices based on nanowire arrays (NW) is considerably lower (0.63%) by comparison, mainly due to low light harvesting ability of ZnO nanowire films. The dye loading of nanowire films is found to be approximately an order of magnitude lower than that of nanoparticle-based ones, regardless of their internal surface area. Inefficient anchoring of dye molecules on the semiconductor surface due to repelling electrostatic forces is identified as the main reason for this low dye loading. We propose a method of modifying the sensitizer solution by altering its pH, thereby enhancing dye adsorption. We report an increase in the PCE of nanowire DSSCs from 0.63% to 1.84% as a direct result of using such a modified dye solution.

## 1. Introduction

Dye-sensitized solar cells (DSSCs) are regarded as a promising option to conventional solid-state semiconductor solar cells due to their low cost, ease of fabrication and environmental friendliness. DSSCs sensitized with Ruthenium-based complexes have achieved maximum power conversion efficiency (PCE) of 11.9% [1,2]. Alternatively, DSSCs with light-harvesting donor–π–acceptor (D–π–A) dyes have yielded efficiencies over 12% [3,4], while various molecularly-engineered porphyrin dyes have been synthesized [5] and resulted in a record 13% efficiency [6]. 

In DSSCs, the conversion of visible light to electricity is achieved through the spectral sensitization of wide bandgap semiconductors such as TiO_2_, ZnO, SnO_2_, NiO, etc. [7,8,9]. ZnO has attracted a great deal of interest in DSSCs applications, as a semiconductor with a wide band gap of 3.37 eV and a high exciton binding energy of 60 meV. In addition, ZnO has high room temperature carrier mobility (115–155 cm^2^ V^−1^ s^−1^), which enhances the performance of solar cells by reducing electron recombination [10].

Typically, the photoelectrode of DSSCs consists of a porous network of semiconductor nanoparticles. The porous nature of the photoelectrode provides a large internal surface area for chromophore anchoring to maximize dye loading (dl) and in turn, light absorption and electron generation [11]. However, the photogenerated electrons then diffuse through the nanoparticle network and will have to travel via a random path also crossing over the grain boundaries before they are collected. This electron transfer process is slow and electrons may interact with traps as they travel through the nanoparticle network and recombine with oxidizing species, thus reducing device efficiency [12,13].

DSSCs based on semiconductor nanowires are considered to have improved electron transport properties by providing a direct pathway for photogenerated electrons from the point of injection to the collecting electrode [14,15,16]. However, the overall PCE of such devices remains relatively low compared to nanoparticle-based ones. The reasons for the low PCE of nanowire-based DSSCs are not fully understood, but the main reason is considered to be reduced light harvesting due to low dye loading [17,18], which was also confirmed by our own findings.

Understanding the mechanisms that affect PCE in nanowire-based DSSCs is key to improving their efficiency. Towards this end, the main parameters that affect device performance, such as semiconductor morphology, light-harvesting efficiency, electron injection and electron collection, were investigated. A comparative assessment of the properties of nanoparticle and nanowire-based DSSCs was conducted to identify the underlying causes of the low nanowire-DSSC performance and improve their efficiency.

## 2. Methodology

### 2.1. ZnO Nanoparticle Films

Nanostructured ZnO films were prepared as described in [19]. Commercial ZnO nanopowder (Sigma-Aldrich, St. Louis, MO, USA) with nanoparticle diameter less than 50 nm was used to create a colloidal paste. The powder was mixed with a small amount of distilled water containing acetyl acetone (10% *v/v*) [20,21] to prevent the coagulation of nanoparticles and improve the porosity of the film [22]. A small amount of Triton X-100 was added to the mixture to reduce surface tension and enable even spreading of the paste [23,24].

The semiconductor oxide paste was spread on conductive glass substrates (K-glass (SnO_2_:F) with sheet resistance 16.7 Ω/sq, 80% transmittance in the visible, 0.38 cm glass thickness) via a doctor blade technique and the electrodes were annealed for 30 min at 400 °C in air to enhance the electrical contact between the nanoparticles as well as between the nanoparticles and the conductive substrate [25]. The thickness of the resulting films was measured by a stylus XP-1 Ambios Technology profilometer. As the thickness of the films can affect the performance of the DSSCs, the photovoltaic characteristics of all nanoparticle-based DSSCs presented in the paper were obtained for samples of approximately the same thickness (ranging from 7 to 10 μm) [26]. We have found in our previous work [26] that ZnO films of approximately this thickness yield the optimum results in terms of device efficiency and stability. The surface area of the ZnO films was approximately 0.5 cm^2^. All films had an oblong shape, with smaller width and greater length. This shape has been shown to be the most favorable for minimizing device internal series resistance and enhancing cell efficiency [27].

### 2.2. ZnO Nanowire Arrays

Nanostructured films based on ZnO NW arrays were developed on glass conductive substrates using wet-chemistry methods. A thin (~10 nm) ZnO seed layer was deposited by spin coating of a solution of 0.05 M Zinc acetate dihydrate (ZnAc, Zn(CH_3_COO)_2_∙2H_2_O) in ethanol at a speed of 1000 rpm followed by annealing at 300 °C for 2 h. Two different approaches were employed for the growth of the NW arrays. In the first one, NW-based films were grown in an aqueous solution at 95 °C containing equal concentrations of ZnAc and hexamethylenetetramine (HMTA, C_6_H_12_N_4_). The concentrations varied to control the NW morphology. NW arrays with narrow to moderate size distributions, and height in the range 0.5–2 μm were prepared for growth times of ~2.5 h. Renewal of the nutrient solution is necessary to achieve NW lengths up to 6–7 μm. Growth of NW arrays with larger diameter and length was also performed using the procedure described in detail elsewhere [28]. After the growth, the NW arrays were rinsed with 3D water and were heated at 300 °C for 2 h to remove undesired contamination from the surface.

The morphologies of the deposited materials were characterized by Field Emission Scanning Electron Microscopy (FE-SEM) (Zeiss SUPRA 35VP-FEG, Jena, Germany) operating at 5–20 keV. The crystal structures of the composites were investigated using X-ray diffraction (XRD) Bruker D8 diffractometer, operating at 40 kV and 40 mA, employing Cu Kα radiation (λ = 1.54056 Å).

### 2.3. Film Sensitization-Assessment of Dye Loading and of the Point of Zero Charge

ZnO films were sensitized in 0.3 mM solutions of the commercial dye N719 (Sigma-Aldrich, St. Louis, MO, USA) in methanol. Coating of the semiconductor surface with the dye was conducted by soaking the film in the dye solution for a time ranging from 30 min to 24 h to optimize the dying process. The optimum time the film should remain in the dye solution was investigated for all types of ZnO films considered to achieve maximum dye adsorption and device efficiency. This was found to depend on film thickness, with thicker films having to remain in the dye solution longer to achieve maximum dye loading. 

Dye loading of the sensitized ZnO films was calculated by desorbing the dye in a 10 mM KOH aqueous solution and then measuring the absorbance of the solution using a UV-Vis PerkinElmer Lambda 650 spectrophotometer (Perkin Elmer, Waltham, MA, USA) [29]. The pH of the dye solution was measured using a Mettler Toledo FE20/FG2 pH Meter (Mettler Toledo, Columbus, OH, USA). The pH of the point of zero charge (pH_PZC_) of ZnO films was obtained using the pH-drift method [30,31]. The point of zero charge was determined by varying the pH of a 0.01 M NaCl 50 mL solution to pH values in the range of 2 to 12 by adding either HCl or NaOH. A semiconductor film was dipped in the solution and was then left for 48 h to reach an equilibrium pH. For each pH value, the initial pH and the final pH (after 48 h) were measured and plotted against each other. The pH at which the curve crosses the line of pH_final_ = pH_initial_ is the pH of the point of zero charge of the film.

### 2.4. DSSC Fabrication

Counter electrodes were prepared through a three-electrode electrodeposition of an aqueous hexachloroplatinic acid solution (0.002 M) on conductive glass substrates. The electrodeposition was performed using a computer-controlled Function Generator (AMEL, 586) (586, Milano, Italy), a potentiostat–galvanostat (AMEL, 2053) (2053, Milano, Italy) and a noise reducer (AMEL NR 2000) (NR 2000, Milano, Italy).

The sensitized semiconductor electrode and the platinized counter electrode were sealed together using the thermoplastic Surlyn^®^ (DuPont, Wilmington, DE, USA) silicon. A liquid electrolyte was then inserted in the space between the two electrodes. The EL-HPE High Performance Electrolyte (Dyesol) (Queanbeyan, Australia) was used for all samples. The electrolyte was inserted into the cell with a syringe through a small aperture and the cell was then sealed with silicone. Several different devices were prepared and tested for each type of DSSCs presented in this work to ensure the reproducibility and validity of the results.

### 2.5. Device Characterization and Testing

Current–Voltage (I–V) curves of the cells were obtained using a Newport 96000 solar simulator (96000, Irvine, CA, USA) fitted with an AM1.5G filter (Newport AM 1.5G, Irvine, CA, USA) in conjunction with a Keithley 236 source meter. The incident irradiance was measured by a photo diode calibrated against a Melles Griot 13PE001 Broad Band Power Meter (13PE001, Rochester, NY, USA).

The main characteristics of the solar cells can be obtained from these I–V curves, namely the open circuit voltage (*V_oc_*), the short circuit current (*I_sc_*), the fill factor (*FF*) and the efficiency (*η*) of the cell. 

The fill factor of each cell can be calculated according to the following equation:

(1) $$FF=\frac{I_{m}V_{m}}{I_{SC}V_{OC}}$$

where *I_m_* and *V_m_* are the values of the current and the voltage for the maximum power point respectively. Finally, the energy conversion efficiency of each solar cell can be calculated using the following equation:

(2) $$\eta =FF\;\frac{I_{SC}V_{OC}}{S\;G_{T}}$$

where *S* is the surface area of the cell and *G_T_* is the incident light intensity.

Dark current measurements were also conducted for all DSSCs considered applying a bias voltage ramp starting from 0 V and exceeding the open circuit voltage of the cell [32]. Dark current mainly arises when triiodide ions from the electrolyte draw electrons from the semiconductor, reducing the triiodide to iodide. This occurs at the semiconductor/electrolyte interface, when there is no sensitizer adsorbed on the semiconductor surface. A secondary source of dark current is the reduction of the oxidative species of the electrolyte by the glass conductive surface. This can occur if there are pathways for the electrolyte to penetrate through the semiconductor film and reach the glass conductive surface [33]. Regardless of its origin, dark current causes electron recombination and results in the loss of photocurrent [34]. The production of dark current in a cell is also directly linked to its open circuit voltage, with high dark current reducing the open circuit voltage of the cell [35].

Open-circuit voltage decay (OCVD) measurements were conducted by stopping the illumination of the cells under open-circuit conditions and using a potentiostat–galvanostat (AMEL, 2053) to monitor the resulting decline of *V_oc_* [36]. Electron lifetime (*τ_n_*) was then determined by the reciprocal of the derivative of the decay curves normalized by the thermal voltage, using the following equation [33]:

(3) $$\tau_{n}\;=\;-\;\frac{k_{B}\;T}{e}\;{\left(\frac{dV_{oc}}{dt}\right)}^{-1}$$

where *k_B_* is the Boltzmann constant, *T* is the absolute temperature, *e* is the positive elementary charge, and *dV_oc_/dt* is the derivative of the transient open-circuit voltage.

Incident photon to current efficiency (IPCE) spectra were obtained using a Newport setup with a 150 W Xe-lamp and a Newport (Oriel Cornerstone) monochromator. IPCE corresponds to the number of electrons measured as photocurrent in the external circuit divided by the monochromatic photon flux that strikes the cell. 

The IPCE factor is given by the following equation:

(4) $$IPCE\;(\%)\;=\;\frac{1240\;[eV\;nm]\;\times \;J_{ph}\;[mA\;cm^{-2}]}{\lambda \;[nm]\;\times \;\Phi \;[mW\;cm^{-2}]}\times 100$$

where *J_ph_* is the short-circuit photocurrent density for monochromatic irradiation and *λ* and *Φ* are the wavelength and the intensity, respectively, of the monochromatic light [37,38]. 

## 3. Results and Discussion

### 3.1. Film Morphology

**Figure 1:**
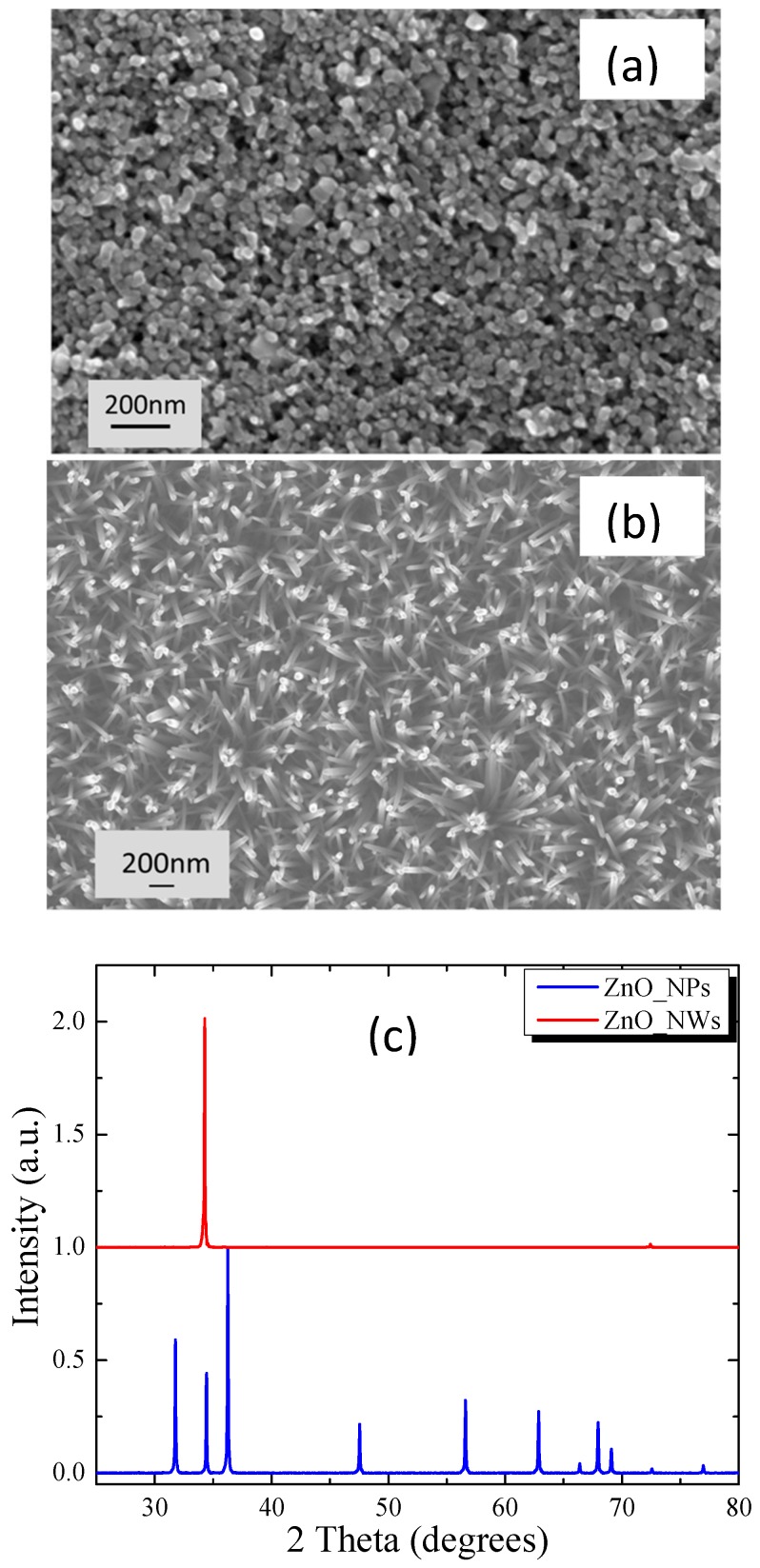
SEM images of: (**a**) ZnO nanoparticle film; and (**b**) a ZnO nanowire film; and (**c**) XRD patterns of a ZnO nanoparticle and a nanowire film.

Figure 1 shows representative SEM images illustrating the morphology of a nanoparticle-based film (Figure 1a) and a nanowire-based film (Figure 1b). The nanoparticle film exhibits high porosity and very low particle agglomeration. The ZnO NWs exhibit high degree of orientation. The XRD patterns of these films are shown in Figure 1c. The length of the ZnO NWs is approximately 7–10 microns, i.e., several orders of magnitude larger than the thickness of the seed layer, which is only 10–20 nm. Therefore, the scattering intensity of the seed layer was considered negligible when obtaining the XRD patterns. The XRD data reveal that ZnO nanowires are highly crystalline and confirm the orientation of these structures normal to the substrate as only the 002 Bragg peak is visible in the corresponding XRD pattern.

**Figure 2:**
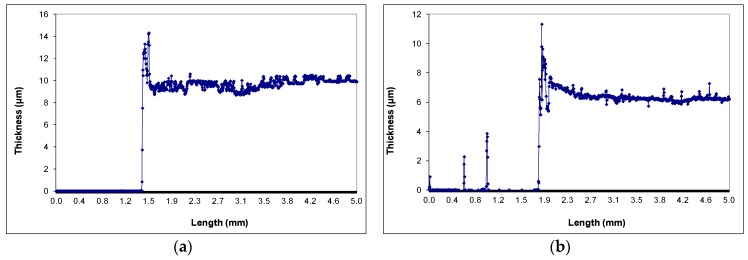
Morphology and thickness of nanowire based films: (**a**) ZnO nanoparticle film; and (**b**) ZnO nanowire film.

Figure 2 shows step profilometer images illustrating the difference in roughness between nanowire and nanoparticle films. The thickness of nanoparticle and nanowire films is also shown in this figure. It can be observed that the film thickness of the nanoparticle films (Figure 2a) is approximately 10 μm. NW films of varying thickness were prepared and tested, by modifying the NW growth conditions. The NW array shown in Figure 2b has been prepared after renewing the nutrient solution two extra times after the initial growth.

**Figure 3:**
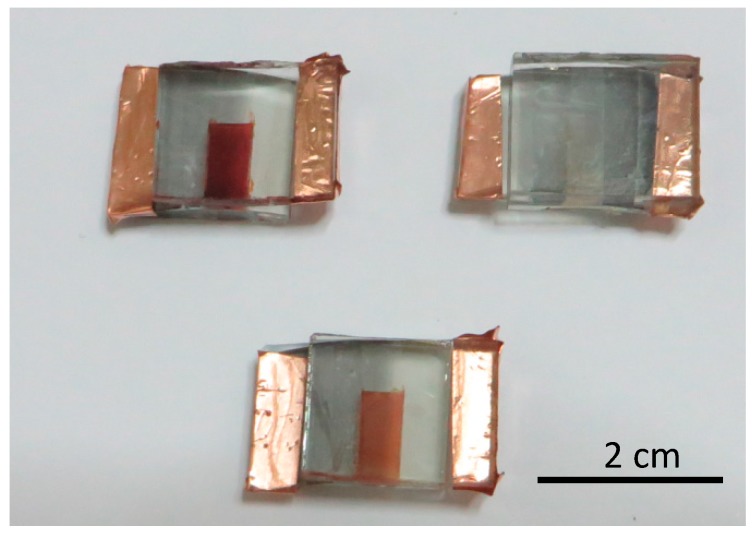
Images of nanoparticle (top left), nanowire (top right) and nanowire with improved dye loading (bottom) devices sensitized with N719.

Figure 3 shows pictures of nanoparticle and nanowire-based devices sensitized with N719. Nanoparticle films sensitized with N719 have a dark red color as expected. Nanowire films on the other hand, have almost no color at all. This effect was common across all samples prepared and tested and it is an indication of very low dye adsorbance on NW films, as confirmed from dye loading measurements. 

### 3.2. Photovoltaic Characteristics

The IPCE of a cell (Equation (4)) can also be expressed in terms of the light-harvesting efficiency of the dye *LHE*(*λ*), the quantum yield of electron injection *η_inj_* and the efficiency of collecting the injected electrons *η_cc_* at the back contact, according to the following equation [39]:

*IPCE*(*λ*) = *LHE*(*λ*) × *η_inj_* × *η_cc_* (5)

**Figure 4:**
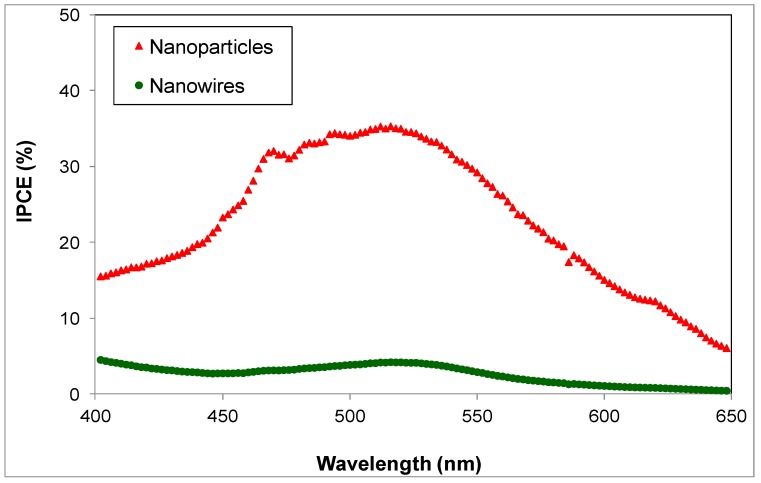
IPCE values of nanoparticle-based and nanowire-based DSSCs as a function of wavelength.

The IPCE values of typical nanoparticle and NW DSSCs are shown in Figure 4 as a function of the illumination wavelength. It is clear from Figure 4 that the IPCE values of nanoparticle DSSCs are considerably higher than those of nanowire cells. These results indicate that one or more of the main parameters that affect IPCE, i.e., light-harvesting efficiency, electron injection and electron collection, has far lower values for NW devices than for nanoparticle ones. In this paper, we will examine the effect of these parameters on the performance of nanoparticle and NW DSSCs and will attempt to identify the factors that are responsible for the low values in the IPCE of NW devices.

**Figure 5:**
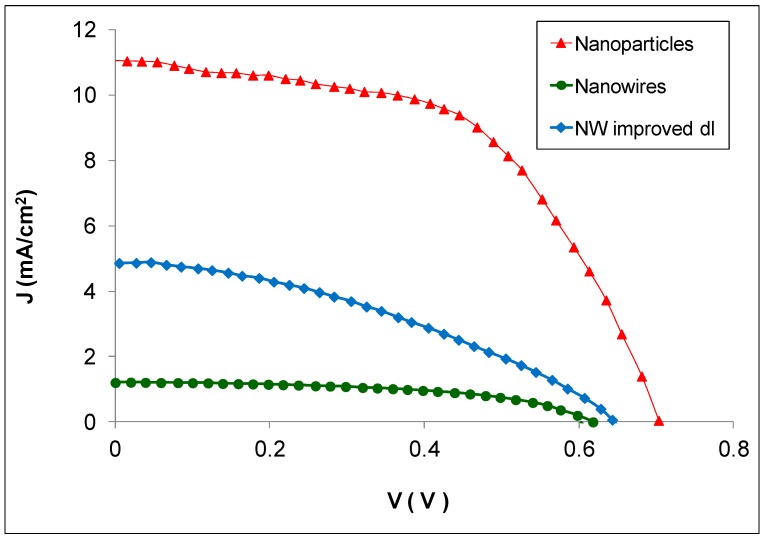
J-V characteristics of ZnO nanoparticle, nanowire and nanowire with improved dye loading DSSCs.

The J-V characteristics of ZnO nanoparticle-based and nanowire-based cells sensitized with the N719 dye are shown in Figure 5 and are summarized in Table 1. Current Density-Voltage (J-V) measurements show that the energy conversion efficiency (*η*) of nanoparticle-based cells sensitized with N719 was an order of magnitude higher than the efficiency of corresponding nanowire-based cells. While ZnO nanoparticle-based DSSCs yielded efficiencies over 6%, nanowire-based devices only reached PCEs of approximately 0.6%. These results are consistent with the large difference between the IPCEs of nanoparticle and NW DSSCs shown in Figure 4. In general, the short-circuit current produced by all nanowire-based devices was considerably lower than that of nanoparticle ones and their open circuit voltage was also lower. Many nanowire-based DSSCs (around 30) have been developed and tested, with varying conditions such as film thickness, time spent in the dye solution, etc. However, even with optimized device conditions, the maximum PCEs achieved were a little over 0.6%. 

The efficiencies reported in this study for nanoparticle-based devices are quite high compared to corresponding values found in the literature. Keis et al. [40] report efficiencies of up to 5% for ZnO nanoparticle films sensitized with N719, while Chang et al. [41] report efficiencies of up to 5.6% for optimized devices with similar properties. He et al. [42] have obtained higher efficiencies (8%) by using air plasma to develop ZnO nanostructures with reduced charge carrier recombination. For NW-based DSSCs, Law et al. [13] report efficiencies of up to 1.5%, while Xu et al. [17] have obtained efficiencies of up to 2.1% by developing optimized ZnO NWs with length of up to 30 μm. While these efficiencies are higher than those reported in the present study, they are still lower than corresponding results for nanoparticle-based devices. The reasons for the low performance of nanowire-based DSSCs compared to nanoparticle ones are not fully understood. It has been reported in the literature that NW cells tend to have lower PCE than nanoparticle-based cells because their overall surface area does not permit high dye loading [17]. Other studies also indicate that nanowire-based DSSCs have lower *FF* than nanoparticle-based ones, due to charge recombination at the interface between the nanowires and the electrolyte [43]. In this study, the mechanisms responsible for the low PCEs of ZnO nanowire DSSCs were investigated to understand the underlying issues that prevent these devices from achieving efficiencies close to those of nanoparticle-based DSSCs. 

**Figure 6:**
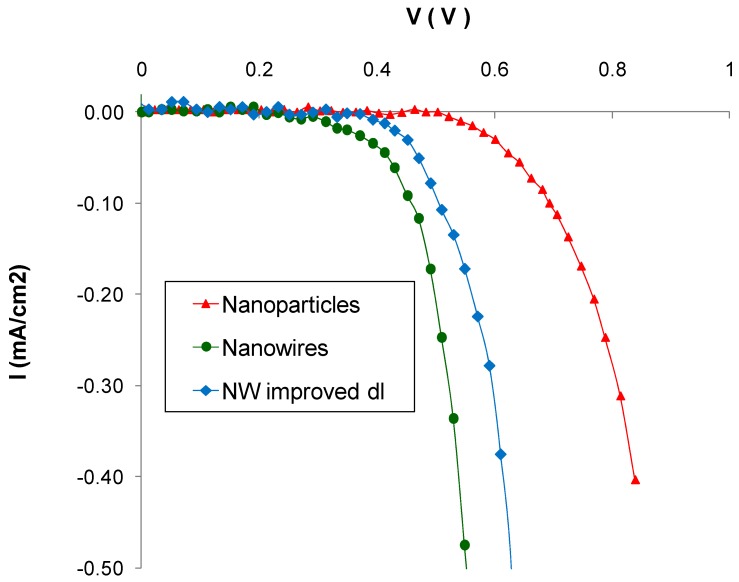
Dark current–voltage characteristics of ZnO nanoparticle, NW and NW with improved dye loading DSSCs.

Figure 6 shows typical dark current–voltage characteristics of nanoparticle-based cells and nanowire-based cells. For NW devices, the onset of dark current is at approximately 0.3 V and its value increases rapidly with increasing voltage. For nanoparticle-based films, on the other hand, the onset of dark current does not occur until approximately 0.55 V. The lower open circuit voltage of the nanowire DSSCs (Figure 5) is probably due to their higher dark current values. 

As mentioned in Section 2, one of the causes of high dark current is the presence of semiconductor sites without dye adsorbed, that come in direct contact with the electrolyte. From Figure 3 it is apparent that dye adsorption in nanowire-based cells is not as successful as in nanoparticle ones and that is probably one of the reasons for the higher dark current of nanowire-based DSSCs. In addition, due to the porous nature of nanowire films, it is possible that there are more pathways for the electrolyte to come into contact with the glass substrate and cause charge recombination, as will be discussed below [44].

**Figure 7:**
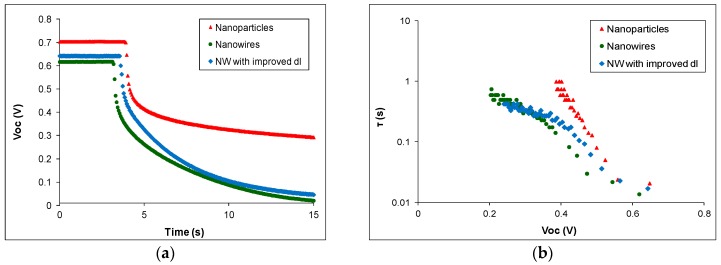
OCVD measurements for ZnO nanoparticle, NW and NW with improved dye loading DSSCs: (**a**) open-circuit voltage decay curves and (**b**) electron lifetime as a function of open-circuit voltage.

OCVD measurements can be used to determine electron recombination rates and ultimately electron lifetimes in DSSCs [45,46]. OCVD experiments measure the recombination rate of electrons injected into the conduction band of the semiconductor. Thus, the photovoltage decay measured reflects the decrease of electron concentration at the conductive glass surface, caused by charge recombination. Figure 7a shows the OCVD decay curves of typical nanoparticle and nanowire-based devices. It is apparent that the rate of open-circuit voltage decay is higher for NW devices than for nanoparticle ones. The electron lifetime with respect to the open-circuit voltage (calculated according to Equation (3)) is presented in Figure 7b and it was found to be higher for nanoparticle-based DSSCs than for nanowire cells. The results shown in Figure 7 indicate that there is higher electron recombination for nanowire DSSCs than for nanoparticle ones. It could be attributed to poor dye loading and possibly to contact of the conductive substrate with the electrolyte, as in the case of dark current. 

The dark current and OCVD results (shown in Figure 6 and Figure 7 respectively) indicate that there are higher carrier losses for NW devices due to electron recombination. These results suggest that reduced electron collection is at least partly responsible for the low performance of NW DSSCs. It has been reported elsewhere [13] that electron injection from the dye’s LUMO to the semiconductor is faster for NW DSSCs sensitized with N719 than for nanoparticle ones. It is therefore clear that the electron injection rate is not the cause of the low efficiency and IPCE of nanowire DSSCs. Below, we study the final reason for low device IPCE, namely the light-harvesting efficiency of DSSCs.

### 3.3. Dye Loading

**Figure 8:**
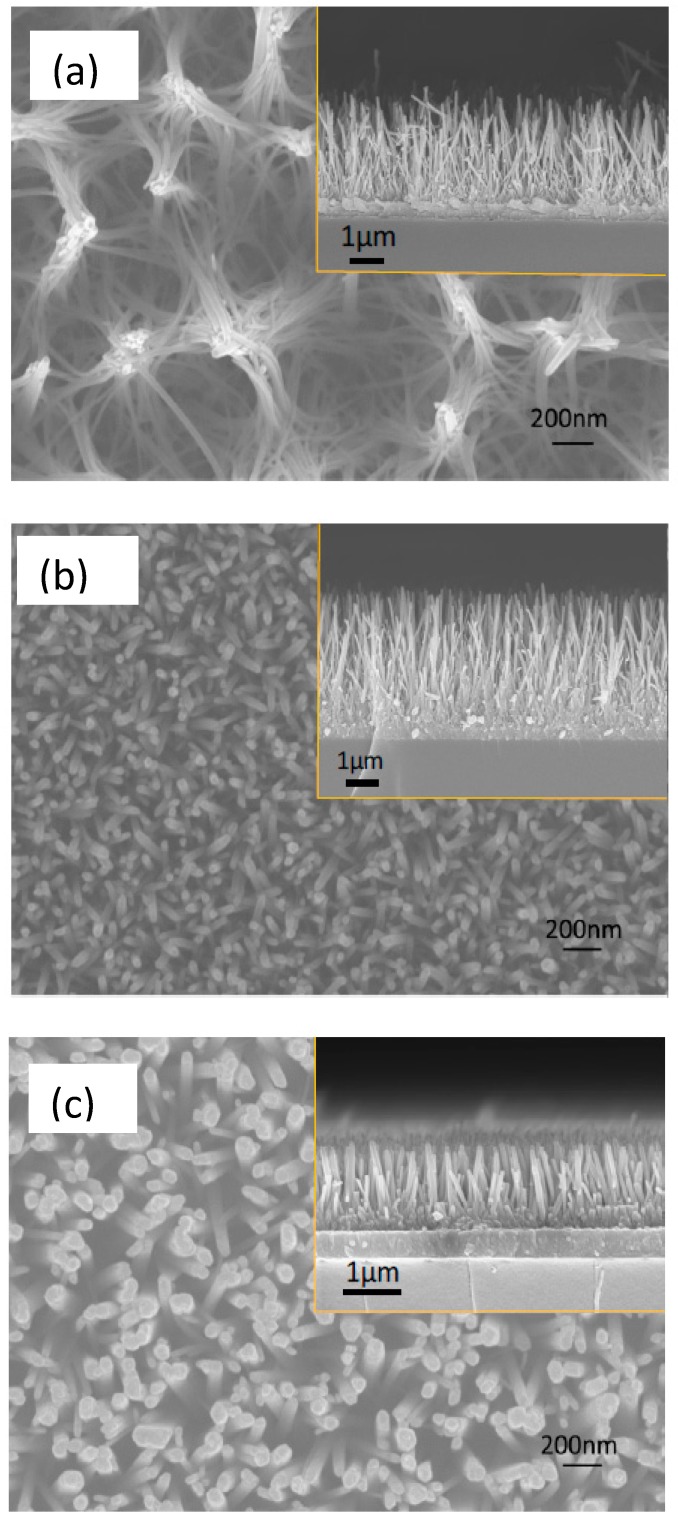
FE-SEM images of ZnO NW arrays used to optimize the dye loading conditions. NWs in (**a**–**c**) have average diameter 20–25, 30–40 and 80–100 nm, respectively.

Dye loading measurements were conducted, as described in Section 2, to assess the amount of dye adsorbed on each type of film. The dye loading was calculated per unit volume (film thickness × film area) to account for the difference in the surface area of the various films. However, the dye loading of the NW films was found to vary considerably depending on film morphology. NW films of different morphologies (Figure 8) were prepared and tested to optimize the dye loading process. The NW films were developed as described in [28] and each type of film was prepared according to the conditions described in Table 2.

**Figure 9:**
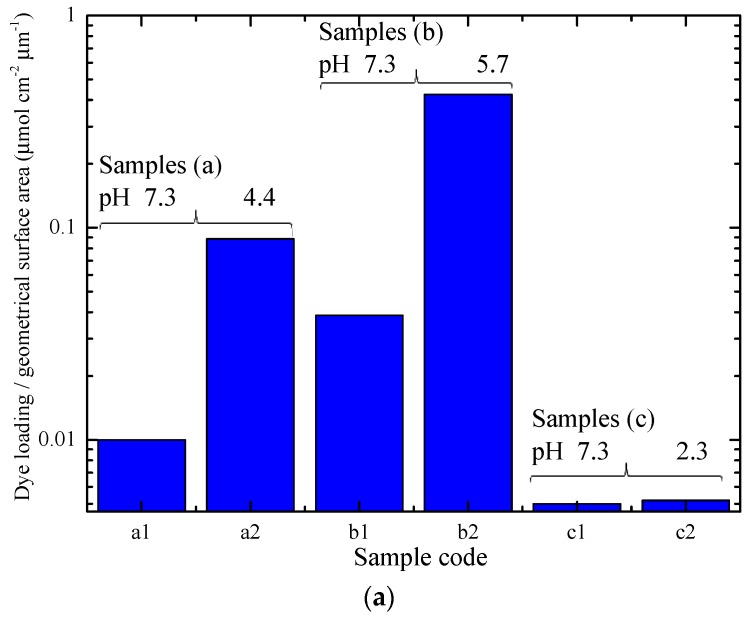

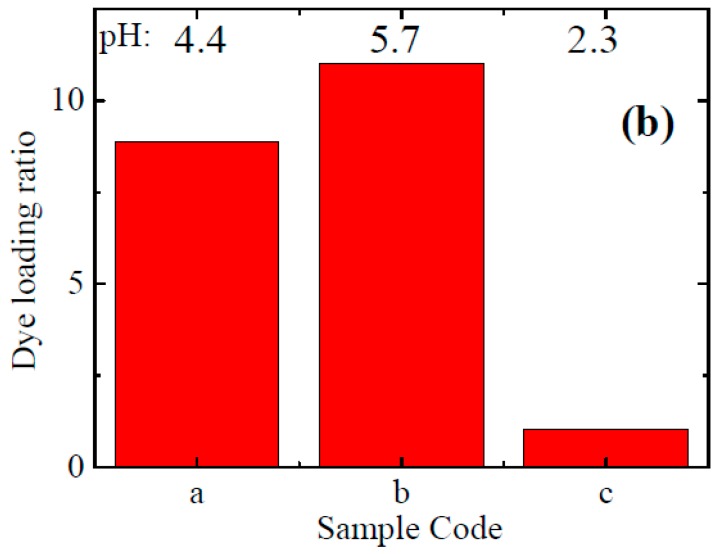
(**a**) Dye loading of NW arrays with the morphology shown in Figure 8. (**b**) Dye loading ratio of twin samples between the selected pH and the pH 7.3.

The dye loading of the various NW films soaked in the standard N719 dye solution (samples a1, b1 and c1) is shown in Figure 9a. The dye loading of nanoparticle films was found to be approximately an order of magnitude higher than that of the nanowire ones with the highest dye loading measured (samples a1 and b1). This difference in the dye loading of nanoparticle and nanowire films accounts for the difference that was observed in the short-circuit current of the two types of devices and is also largely responsible for the difference in their efficiencies. The difference in the dye loading of the two types of films was also expected from visual observations of the films (Figure 3). 

The relatively low dye loading of nanowire films compared to that of nanoparticle ones has also been reported elsewhere [17]. However, the reasons for the low dye absorbance of these films remain unclear. The main difference between the two types of films lies in the morphology of the ZnO nanostructures, which can also affect their surface charge. The adsorption mechanism of N719 on a ZnO surface has been shown to be predominantly electrostatically driven [47]. It has also been reported [47,48] that N719 forms more stable bonds with semiconductor films in a protonated environment. If the semiconductor surface is negatively charged on the other hand, it may repel the carboxylic groups in N719 and the dye may not be adsorbed on the semiconductor surface successfully. 

**Figure 10:**
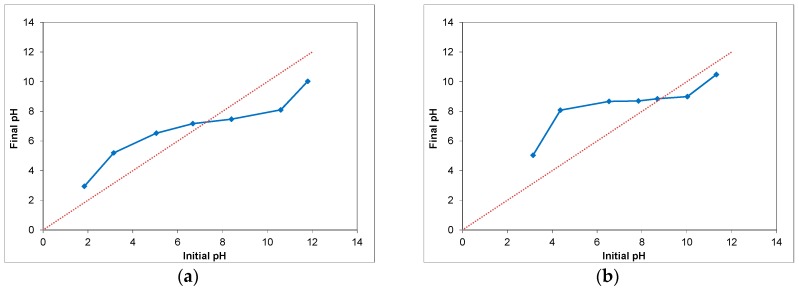
Determination of the pH_PZC_ of ZnO films: (**a**) nanowires; and (**b**) nanoparticles.

To test this hypothesis, the pH of the point of zero charge (pH_PZC_) of ZnO nanowire films was determined as described in Section 2.3 and compared to the pH_PZC_ of ZnO nanoparticle films. The values of the final against the initial pH are shown in Figure 10 and the pH at which the curve crosses the line of pH_final_ = pH_initial_ is the pH of the point of zero charge of the film. In this case, the pH_PZC_ of ZnO nanowires (Figure 8a) was found in the range of 6.7–7.2 (the range represents the results of pH_PZC_ measurements in different samples). The pH_PZC_ of ZnO nanoparticles was found to be approximately 8.9 (Figure 8b), which agrees with corresponding results reported in the literature [49]. 

The high value of the pH_PZC_ of ZnO nanoparticles indicates the ZnO nanoparticle surface is positively charged, which may facilitate the adsorption process. The same is not true for ZnO nanowire films however, which may hinder the anchoring process of the dye on the semiconductor surface. The pH of the standard dye solution used was measured to be in the range of 7.3–7.7 (the range represents the pH measurements in various dye solutions), which is slightly higher than the pH_PZC_ of ZnO nanowire films. When a nanowire film is submerged in the standard dye solution, the net electrical charge on the film surface will be negative. On the other hand, the pH_PZC_ of ZnO nanoparticle films is higher than the pH of the standard dye solution, which means that the nanoparticle film/dye solution environment will be protonated. The same result can be achieved for the nanowire film/dye solution environment by lowering the pH of the dye solution below the pH_PZC_ of nanowire films. Acidic dye solutions will have a higher concentration of positive ions, which may facilitate the adsorption process for ZnO nanowire films.

To this aim, three pairs of ZnO NW films were prepared with similar morphology for each pair as shown in Figure 8. Three different pH values, i.e., 2.3, 4.4 and 5.7, were selected. One sample of each pair was soaked for 2 h in N719 solutions with pH 7.3, while the other sample of each pair was soaked for the same period in solutions of N719 with the above pH values. The dye loading of the films was estimated and the results are shown in Figure 9. Since a comparison between films with vastly different morphologies, as those shown in Figure 8, may lead to misleading conclusions we undertake only a comparison between the twin samples, i.e., one soaked in pH 7.3 and the corresponding one soaked in one of the other pH values. Figure a shows the dye loading of the samples with morphologies of type (a), (b), and (c) corresponding to the images of Figure 8. The data in Figure 9a are normalized with respect to the NW length and sample surface area. For moderate acidic pH (4.4 and 5.7), the dye loading was considerably improved compared to that at pH 7.3. The films soaked in the dye solution with PH equal to 4.4 and 5.7 were also more intensely colored, as shown in Figure 3. For the more acidic solution, i.e., at pH 2.3, there was no significant change in the dye loading with respect to that at pH 7.3. 

Figure 9b shows the ratio of the dye loading achieved at the acidic pH in relation to the normal pH for all three types of NW morphologies. The gain in dye loading for the samples soaked in the solutions with pH values 2.3, 4.4 and 5.7 are ~1, ~9, and ~11, respectively. These data show best performance for pH 5.7, while even the more acidic solution (pH 4.4) still provides improved dye loading. The dye loading of nanoparticle films, however, remained higher than that of the best performing NW films (at pH 5.7) by approximately 20%. 

In the case of very acidic dye solutions (pH 2.3) it was observed that there is rapid (within a few hours) degradation of the dye solution in the presence of ZnO films. Dye degradation in ZnO films occurs due to protons originating from the dye interacting with the ZnO surface and causing the dissolution of Zn surface atoms [50]. The Zn atoms then react with the protons from the dye, forming Zn^+2^/dye complexes. This leads to the formation of inactive dye molecules which limit charge carrier injection and reduce the efficiency of the cells. The dissolution of Zn atoms depends on several factors, such as the dye concentration, the sensitization time and the pH of the dye solution [49]. Thus, to achieve optimum dye loading and efficiency, the pH of the dye should be lower than the pH_PZC_ of the film, but not so low that it causes dye degradation. It should also be noted that the samples of type (c) had lower dye loading at pH 7.3 than the samples of the other two types. This illustrates the effect of film morphology on the dye loading of the films. It can be observed from Figure 8 that the samples of type (c) had NWs with a diameter up to five times as large as that of the two other sample types. The higher diameter of the NWs leads to the formation of films with lower surface area for dye adsorption and, consequently, lower dye loading. 

### 3.4. Nanowire DSSCs with Improved Dye Loading

The J-V characteristics of NW DSSCs with improved dye loading (pH of dye solution equal to 5.7) are shown in Figure 5, compared with those of nanoparticle-based cells and standard NW ones. The photovoltaic properties of these cells are summarized in Table 1. It is apparent from Figure 5 that the overall performance of NW cells with improved dye loading is considerably higher than that of the standard pH dye loading of NW cells. The *V_oc_* of devices with improved dl is only slightly higher than that of the standard devices and it is still lower than the *V_oc_* of the nanoparticle DSSCs. The *J_sc_* of the devices with improved dl however, is almost four times higher than that of standard ones and that can be attributed directly to their increased dye loading. Their efficiency is also almost three times higher than the efficiency of standard nanowire DSSCs. It should be noted here that many devices of each type (approximately 30 NW, 30 nanoparticle DSSCs and over 10 NW with improved dye loading conditions) were prepared and tested to ascertain the validity of our results, and that the results shown here are representative of each type of DSSC considered. The error values shown in Table 1 reflect the variation in the J-V measurements across samples of the same type. These error values indicate that there is a high degree of reproducibility across all samples prepared and tested. 

Although improving the dye loading of the devices resulted in a considerable increase in the efficiency of NW DSSCs, their performance is still not as high as that of nanoparticle devices. The dye loading of nanowire films is still lower than that of nanoparticle ones and that results in lower device current density. In addition, there are indications of high electron recombination in the case of nanowire DSSCs, as will be shown below.

Figure 6 and Figure 7 show dark current and OCVD results, respectively, for each type of cell considered. Figure 6 indicates that the dark current of NW devices with improved dl is slightly lower compared to that of standard ones, but it is still quite high compared to that of nanoparticle DSSCs. The reduction in dark current is probably due to improved dye adsorption, which leads to lower electron recombination at the interface between the semiconductor and the electrolyte. However, the OCVD results of Figure 7 show that NW devices with improved dl have similar electron lifetimes with standard ones, which in both cases are lower than those of ZnO nanoparticles. This indicates that there is still high electron recombination, possibly due to bare sites on the glass substrate. This is understandable due to the more porous nature of the nanowire films. Their seed layer is only a few nm thick and it can be more easily permeated by the electrolyte in the gaps between the nanowires. By contrast, nanoparticle films consist of a layer of a few μm thick, which is harder for the electrolyte to penetrate.

## 4. Conclusions

A systematic study of the parameters that affect the performance of nanowire-based DSSCs was conducted to investigate the underlying issues that prevent these devices from achieving PCEs close to those of nanoparticle-based DSSCs. Decreased light harvesting efficiency due to low internal surface area has been broadly considered to be the main reason for the low PCE of nanowire-based DSSCs. Here, an attempt was made to further explain the reasons for the low performance of these devices. To this aim, the dye loading of nanoparticle and nanowire films was measured, regardless of their surface area, and it was found that the dl of nanoparticle films is approximately an order of magnitude higher than that of nanowire ones, thus largely accounting for the higher short-circuit current and PCE of nanoparticle DSSCs.

pH drift measurements were conducted to determine the point of zero charge of nanowire films and it was found that it is somewhat lower than the pH of the dye solution. This may cause the nanowire surface to electrostatically repel dye molecules and prevent them from successfully anchoring to the semiconductor surface. Using acidic dye solutions, which have a higher concentration of positive ions, to sensitize nanowire films led to significantly increased dye adsorption. The dye loading of nanowire films soaked in a solution of N719 with pH equal to 5.7 was found to be higher than that of corresponding films sensitized with the standard dye solution by a factor of approximately 11, and was only 20% lower than the dye loading of nanoparticle films.

The overall performance of nanowire cells with improved dye loading was considerably higher than that of the standard nanowire cells. The short-circuit current of the devices with improved dl was almost four times higher than that of standard ones, which is consistent with the increase in their dye loading. Their efficiency was also almost three times higher than the efficiency of standard nanowire DSSCs.

Electron collection in nanowire DSSCs was expected to be higher than that of corresponding nanoparticle devices, since the morphology of the nanowire films provides direct pathways for electrons to travel from the semiconductor to the collection electrode. From dark current and OCVD measurements, however, it was apparent that nanowire-based DSSCs have higher recombination losses than nanoparticle ones. Electron recombination in DSSCs occurs mainly at the interface between the semiconductor and the electrolyte. Due to the low dye loading of nanowire films, there would be many semiconductor sites without dye adsorbed. These free semiconductor sites could come in direct contact with the electrolyte, leading to a greater chance of electron recombination. Increasing the dye loading of nanowire films was found to lead to a decrease in the dark current, but not to the low levels of nanoparticle-based devices. It is possible that there is high electron recombination through contact of the electrolyte with the glass substrate, due to the more porous nature of the nanowire films.

## Figures and Tables

**Table 1:** Summary of the PV properties of the ZnO DSSCs.

Sample Type Header	*V_oc_* (V)	*J_sc_* (mA/cm^2^)	*FF*	*η* (%)
Nanoparticles	0.70 ± 0.02	11.2 ± 1.0	0.54 ± 0.06	6.19 ± 0.60
Nanowires	0.62 ± 0.03	1.2 ± 0.4	0.53 ± 0.04	0.63 ± 0.09
Nanowires with improved dl	0.64 ± 0.02	4.8 ± 0.4	0.41 ± 0.03	1.80 ± 0.20

**Table 2:** Growth conditions of the three types of samples shown in Figure 8.

Sample Code	Seed Layer	Growth Conditions	
a	0.05 M Zinc acetate in ethanol	0.04 M Zn(NO_3_)_2_ 0.02 M HMTA 0.16 M PEI 0.04 M NH_4_OH	Without renewal
b		0.04 M Zn(NO_3_)_2_ 0.02 M HMTA 0.16 M PEI 0.04 M NH_4_OH	With renewal
c	0.005 M Zinc acetate in ethanol	0.05 M Zn(NO_3_)_2_ 0.025 M HMTA 0.08 gr PEI 0.7 M NH_4_OH	Without renewal

## References

[1] Green M.A., Hishikawa Y., Dunlop E.D., Levi D.H., Ebinger J.H., Ho-Baillie A. (2018). Solar cell efficiency tables (version 51). Prog. Photovolt..

[2] Komiya R., Fukui A., Murofushi N., Koide N., Yamanaka R., Katayama H. Improvement of the conversion efficiency of a monolithic type dye-sensitized solar cell module. Proceedings of the Technical Digest, 21st International Photovoltaic Science and Engineering Conference.

[3] Kanaparthi R.K., Kandhadi J., Giribabu L. (2012). Metal-free organic dyes for dye-sensitized solar cells: Recent advances. Tetrahedron.

[4] Yella A., Lee H.W., Tsao H.N., Yi C., Chandiran A.K., Nazeeruddin M.K., Diau E.W., Yeh C.Y., Zakeeruddin S.M., Grätzel M. (2011). Porphyrin-sensitized solar cells with cobalt (II/III)-based redox electrolyte exceed 12 percent efficiency. Science.

[5] Campbell W.M., Jolley K.W., Wagner P., Wagner K., Walsh P.J., Gordon K.C., Schmidt-Mende L., Nazeeruddin M.K., Wang Q., Grätzel M. (2007). Highly Efficient Porphyrin Sensitizers for Dye-Sensitized Solar Cells. J. Phys. Chem. C.

[6] Mathew S., Yella A., Gao P., Humphry-Baker R., Curchod B.F., Ashari-Astani N., Tavernelli I., Rothlisberger U., Nazeeruddin M.K., Grätzel M. (2014). Dye-sensitized solar cells with 13% efficiency achieved through the molecular engineering of porphyrin sensitizers. Nat. Chem..

[7] Grätzel M., O’Regan B. (1991). A Low-Cost, High-Efficiency Solar Cell Based on Dye-Sensitized Colloidal TiO_2_ Films. Nature.

[8] Wang Y., Tian J., Fei C., Lv L., Liu X., Zhao Z., Cao G. (2014). Microwave-Assisted Synthesis of SnO_2_ Nanosheets Photoanodes for Dye-Sensitized Solar Cells. J. Phys. Chem. C.

[9] Farré Y., Zhang L., Pellegrin Y., Planchat A., Blart E., Boujtita M., Hammarström L., Jacquemin D., Odobel F. (2016). Second Generation of Diketopyrrolopyrrole Dyes for NiO-Based Dye-Sensitized Solar Cells. J. Phys. Chem. C.

[10] Morkoc H., Ozgur U. (2008). Zinc Oxide Fundamentals, Materials and Device Technology.

[11] Schlur L., Carton A., Lévêque P., Guillon D., Pourroy G. (2013). Optimization of a New ZnO Nanorods Hydrothermal Synthesis Method for Solid State Dye Sensitized Solar Cells Applications. J. Phys. Chem. C.

[12] Kao M.C., Chen H.Z., Young S.L., Lin C.C., Kung C.Y. (2012). Structure and photovoltaic properties of ZnO nanowire for dye-sensitized solar cells. Nanoscale Res. Lett..

[13] Law M., Greene L., Johnson J., Saykally R., Yang P. (2005). Nanowire dye-sensitized solar cells. Nat. Mater..

[14] Cheng H.M., Chiu W.H., Lee C.H., Tsai S.Y., Hsieh W.F. (2008). Formation of Branched ZnO Nanowires from Solvothermal Method and Dye-Sensitized Solar Cells Applications. J. Phys. Chem. C.

[15] Bai Y., Yu H., Li Z., Amal R., Lu G.Q., Wang L. (2012). In situ growth of a ZnO nanowire network within a TiO_2_ nanoparticle film for enhanced dye-sensitized solar cell performance. Adv. Mater..

[16] Yang Z., Xu T., Ito Y., Welp U., Kwok W.K. (2009). Enhanced Electron Transport in Dye-Sensitized Solar Cells Using Short ZnO Nanotips on A Rough Metal Anode. J. Phys. Chem. C.

[17] Xu C.K., Shin P., Cao L.L., Gao D. (2010). Preferential Growth of Long ZnO Nanowire Array and Its Application in Dye-Sensitized Solar Cells. J. Phys. Chem. C.

[18] Guerin V.M., Rathousky J., Pauporte T. (2012). Electrochemical design of ZnO hierarchical structures for dye-sensitized solar cells. Sol. Energy Mater. Sol. Cells.

[19] Giannouli M. (2013). Nanostructured ZnO, TiO_2_, and Composite ZnO/TiO_2_ Films for Application in Dye-Sensitized Solar Cells. Int. J. Photoenergy.

[20] Nazeeruddin M.K., Kay A., Rodicio I., Humpbry-Baker R., Miiller E., Liska P., Vlachopoulos N., Grätzel M. (1993). Conversion of light to electricity by cis-X2bis(2,2′-bipyridyl-4,4′-dicarboxylate)ruthenium(II) charge-transfer sensitizers (X = Cl-, Br-, I-, CN-, and SCN-) on nanocrystalline titanium dioxide electrodes. J. Am. Chem. Soc..

[21] Smestad G. (1998). Education and solar conversion: Demonstrating electron transfer. Sol. Energy Mater. Sol. Cells.

[22] Pichot F., Pitts R., Gregg B. (2000). Low-Temperature Sintering of TiO_2_ Colloids:  Application to Flexible Dye-Sensitized Solar Cells. Langmuir.

[23] Liu Y., Wang H., Shen H., Chen W. (2010). The 3-dimensional dye-sensitized solar cell and module based on all titanium substrates. Appl. Energy.

[24] Van der Zanden B., Goossens A. (2000). The Nature of Electron Migration in Dye-Sensitized Nanostructured TiO_2_. J. Phys. Chem. B.

[25] Shklover V., Nazeeruddin M.K., Zakeeruddin S.M., Barbe C., Kay A., Haibach T., Steurer W., Hermann R., Nissen H.U., Grätzel M. (1997). Structure of Nanocrystalline TiO_2_ Powders and Precursor to Their Highly Efficient Photosensitizer. Chem. Mater..

[26] Giannouli M., Spiliopoulou F. (2012). Effects of the morphology of nanostructured ZnO films on the efficiency of dye-sensitized solar cells. Renew. Energy.

[27] Lee W., Okada H., Wakahara A., Yoshida A. (2006). Structural and photoelectrochemical characteristics of nanocrystalline ZnO electrode with Eosin-Y. Ceram. Int..

[28] Syrrokostas G., Govatsi K., Yannopoulos S.N. (2016). High-Quality, Reproducible ZnO Nanowire Arrays Obtained by a Multiparameter Optimization of Chemical Bath Deposition Growth. Cryst. Growth Des..

[29] Han J., Fan F., Xu C., Lin S., Wei M., Duan X., Wang Z.L. (2010). ZnO nanotube-based dye-sensitized solar cell and its application in self-powered devices. Nanotechnology.

[30] Benhebal H., Chaib M., Salmon T., Geens J., Leonard A., Lambert S.D., Crine M., Heinrichs B. (2013). Photocatalytic degradation of phenol and benzoic acid using zinc oxide powders prepared by the sol–gel process. Alex. Eng. J..

[31] Lopez-Ramon M.V., Stoeckli F., Moreno-Castilla C., Carrasco-Marin F. (1999). On the characterization of acidic and basic surface sites on carbons by various techniques. Carbon.

[32] Giannouli M., Fakis M. (2011). Interfacial Electron Transfer Dynamics and Photovoltaic Performance of TiO_2_ and ZnO Solar Cells Sensitized with Coumarin 343. J. Photochem. Photobiol. A.

[33] Yu H., Zhang S., Zhao H., Will G., Liu P. (2009). An Efficient and Low-Cost TiO_2_ Compact Layer for Performance Improvement of Dye-Sensitized Solar Cells. Electrochim. Acta.

[34] Xu W., Dai S., Hu L., Zhang C., Xiao S., Luo X., Jing W., Wang K. (2007). Influence of Different Surface Modifications on the Photovoltaic Performance and Dark Current of Dye-Sensitized Solar Cells. Plasma Sci. Technol..

[35] Jasim K.H. (2011). Dye Sensitized Solar Cells—Working Principles, Challenges and Opportunities.

[36] Zaban A., Greenshtein M., Bisquert J. (2003). Determination of the electron lifetime in nanocrystalline dye solar cells by photovoltage decay measurements. ChemPhysChem.

[37] Chiba Y., Islam A., Watanabe Y., Komiya R., Koide N., Han L. (2006). Dye-Sensitized Solar Cells with Conversion Efficiency of 11.1%. Jpn. J. Appl. Phys..

[38] Hara K., Tachiban Y., Ohga Y., Shinpo A., Suga S., Sayama K., Sugihara H., Arakawa H. (2003). Dye-sensitized nanocrystalline TiO_2_ solar cells based on novel coumarin dyes. Sol. Energy Mater. Sol. Cells.

[39] Rani S., Shishodia P.K., Mehra R.M. (2010). Development of a dye with broadband absorbance in visible spectrum for an efficient dye-sensitized solar cell. J. Renew. Sustain. Energy.

[40] Keis K., Magnusson E., Lindström H., Lindquist S., Hagfeldt A. (2002). A 5% efficient photoelectrochemical solar cell based on nanostructured ZnO electrodes. Sol. Energy Mater. Sol. Cells.

[41] Chang W., Lee C., Yu W., Lin C. (2012). Optimization of dye adsorption time and film thickness for efficient ZnO dye-sensitized solar cells with high at-rest stability. Nanoscale Res. Lett..

[42] He Y., Hu J., Xie Y. (2015). High-efficiency dye-sensitized solar cells of up to 8.03% by air plasma treatment of ZnO nanostructures. Chem. Commun..

[43] Jiang C., Sun X., Lo G., Kwong D. (2007). Improved dye-sensitized solar cells with a ZnO-nanoflower photoanode. Appl. Phys. Lett..

[44] Ito S., Liska P., Comte P., Charvet R., Pechy P., Bach U., Schmidt-Mende L., Zakeeruddin S.M., Kay A., Nazeeruddin M.K. (2005). Control of dark current in photoelectrochemical (TiO_2_/I--I3-)) and dye-sensitized solar cells. Chem. Commun..

[45] Bisquert A., Zaban M., Greenshtein M., Mora-Seró I. (2004). Determination of Rate Constants for Charge Transfer and the Distribution of Semiconductor and Electrolyte Electronic Energy Levels in Dye-Sensitized Solar Cells by Open-Circuit Photovoltage Decay Method. J. Am. Chem. Soc..

[46] Fabregat-Santiago F., Garcia-Canadas J., Palomares E., Clifford J.N., Haque S.A., Durrant J.R., Garcia-Belmonte G., Bisquert J. (2004). The origin of slow electron recombination processes in dye-sensitized solar cells with alumina barrier coatings. J. Appl. Phys..

[47] Azpiroz J.M., De Angelis F. (2014). DFT/TDDFT Study of the Adsorption of N3 and N719 Dyes on ZnO(101̅0) Surfaces. J. Phys. Chem. A.

[48] Schiffmann F., Van deVondel J., Hutter J., Wirz R., Urakawa A., Baiker A. (2010). Protonation-Dependent Binding of Ruthenium Bipyridyl Complexes to the Anatase(101) Surface. J. Phys. Chem. C.

[49] Bahnemann D.W. (1993). Ultrasmall Metal Oxide Particles: Preparation, Photophysical Characterization, and Photocatalytic Properties. Isr. J. Chem..

[50] Keis K., Bauer C., Boschloo G., Hagfeldt A., Westermark K., Rensmob H., Siegbahn H. (2002). Nanostructured ZnO electrodes for dye-sensitized solar cell applications. J. Photochem. Photobiol. A.

